# Endodontic Treatment of a Large Periradicular Lesion: A Case Report

**Published:** 2008-10-01

**Authors:** Saeed Asgary, Sara Ehsani

**Affiliations:** 1 *Department of Endodontics, Iranian Center for Endodontic Research, Dental Research Center, Dental School, Shahid Beheshti University of Medical Sciences, Tehran, Iran*; 2 *Oral Radiologist, Dental Research Center, Shahid Beheshti University of Medical Sciences, Tehran, Iran*

**Keywords:** Healing, Maxillary Sinus, Office Visits, Radicular Cyst, Root Canal Therapy

## Abstract

This case report describes the endodontic treatment of a large cyst-like periradicular lesion a 29-year-old female with a large chronic periapical abscess in the region of right maxillary sinus presented into private practice, accompanied with non-vital first upper molar and poorly root treated second upper molar. Conservative root canal treatment was carried out for both of the involved teeth in a single appointment. Post operative examination after two weeks revealed complete resolution of the sinus tract. The clinical and radiographic examination after 9 months revealed complete periapical healing. The appropriate diagnosis of periradicular lesion and the treatment of the infected root canal system allowed complete healing of these large lesions without endodontic surgery.

## INTRODUCTION

Pulpal tissue can become infected through various ways such as caries or trauma, making the pulpal tissue necrotic. The microbial aggregation or its by-products can infiltrate into periradicular tissues and stimulate the host defense system, resulting in periapical/ periradicular tissue destruction. Although this defensive lesion may be helpful to prevent further progress of the microbial infection, it is not self-healing and results in various types of lesions (1). The general consensus is that bacterial reduction or elimination from the root canal system by effective biomechanical preparation will lead to more successful outcomes (2). Investigators have shown that large periradicular lesion may respond positively to nonsurgical endodontic treatment (3-5). In cases were response to conservative treatment is not successful other treatment modalities can be considered. Non-surgical retreatment is usually the treatment of choice though occasionally periradicular surgery may be the treatment of choice, or even extraction may be necessary to allow the lesion to heal (4). The following case report describes an orthograde endodontic treatment of first and second maxillary molars associated with a large cyst-like periradicular lesion.

## CASE REPORT

**Figure 1:**
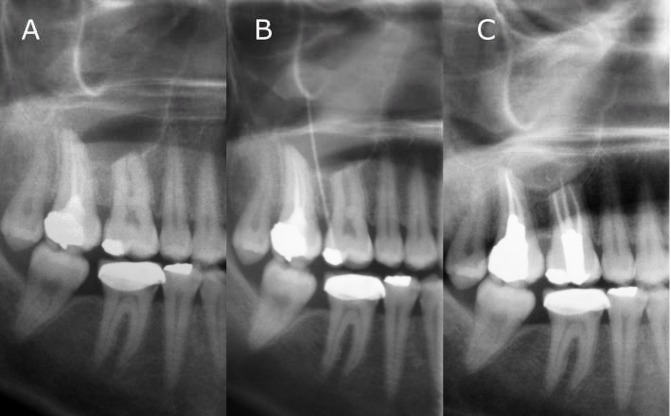
Panoramic radiography demonstrating: A) First and second right maxillary molars probably responsible for the cyst-like lesion appearing at their apices, B) Gutta-percha used for tracing the course of sinus tract showing occluso-apical dimension of the lesion which was not detected in periapical view, and C) Complete healing of the lesion 9 months after root canal therapy and permanent restoration

A 29-year-old female attended a private endodontic clinic; her chief complaint was the presence of mild pain in right maxillary sinus area. The patient had no significant medical history. Right maxillary first molar was not previously root treated, was not carious and had no history of trauma. The adjacent second molar had poor endodontic root treatment with an incomplete obturation (Figure 1-A).

Extra oral examination revealed no sign or symptom. Intraoral examination revealed a minor firm swelling of the vestibule above the molars and an associated sinus tract on the buccal alveolar process. Palpation produced purulent exudates and the mucosa in the region was inflamed. Teeth were not tender to percussion and were not mobile. Electric pulp test and cavity test exhibited negative results for right maxillary first molar.

**Figure 2:**
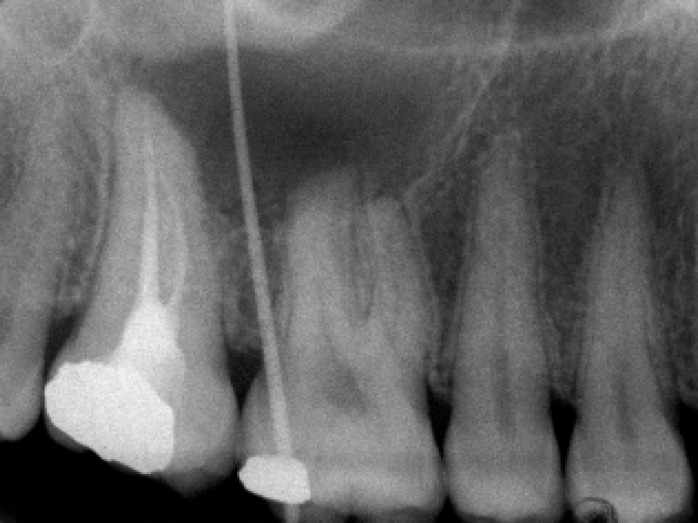
Periapical view showing a part of customized gutta-percha for tracing

Gutta-percha cone was used to trace the path of sinus tract by periapical x-ray (Figure 2); however as the entire course of the sinus tract was not apparent, panoramic radiograph was taken (Figure 1-B). The panoramic tomograoh revealed a well-circumscribed radiolucency measuring approximately 25 mm in diameter, extending from distal aspect of the second premolar to distal aspect of the second maxillary molar. Right maxillary first molar also showed a profound root resorption. Adjacent teeth had no root resorption.

The patient´s clinical and radiographic findings seemed to suggest a large cyst-like peri-radicular lesion, most likely to be an infected radicular cyst of endodontic origin.

**Figure 3:**
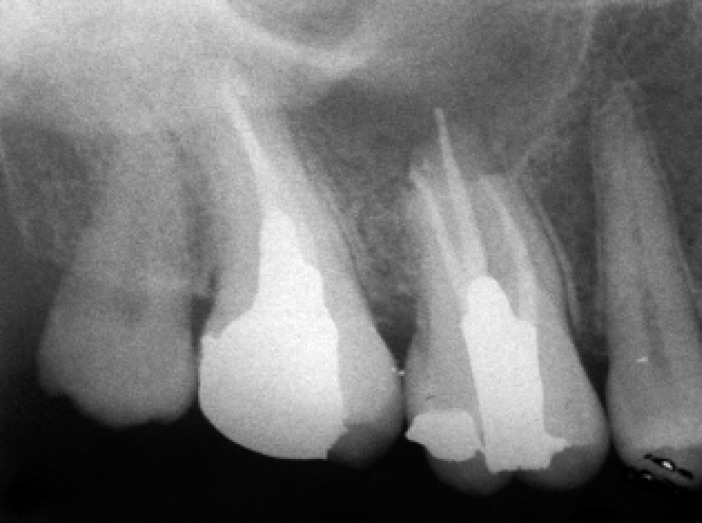
Periapical radio-graph after root canal filling and permanent restoration

One visit endodontic treatment was performed for the right maxillary first and second molars, in one session. After access cavity preparation, treatment was continued with a rubber dam in place. There were no exudates from the canals. Instrumentation was performed by Flexo-File (Dentsply, Maillefer, Switzerland) #15-40, using step-back technique, accompanying with copious irrigation with sterile normal saline between instruments. The working length was determined on the basis of radiographs. Obturation was performed with gutta-percha (Ariadent, Tehran, Iran) and sealer (Roth's 801 sealer, Roth International, Chicago, IL, USA) by lateral condensation technique (Figure 3).

After two weeks of treatment, teeth were permanently restored with amalgam (Synalloy, Dentoria, France). The patient was recalled after one day, two weeks, 9 and 12 months. The signs and symptoms, including the sinus tract, had disappeared after two weeks of treatment.

**Figure 4:**
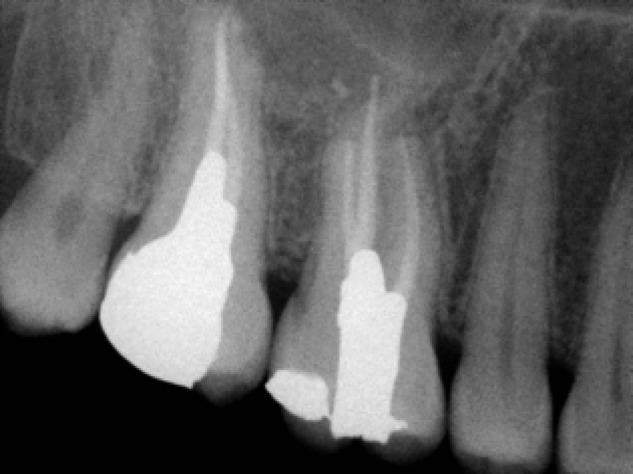
Periapical radio-graph demonstrating healing on the 12-months recall visit

On 9 month and one year recalls, the patient had no sign and symptom; panoramic and periapical radiographic evaluation demonstrate-ed complete bony regression of the lesion (Figure 1-C) and (Figure 4). Clinical exam revealed no sensitivity to percussion and palpation.

## DISCUSSION

This case illustrated a cyst-like periradicular lesion, most probably a radicular cyst. The exact diagnosis can be made by microscopic examination. However, the clinical diagnosis of a radicular cyst seemed rational because the lesion accompanied nonvital teeth, was more than 1.6 mm in diameter, and was bordered with a radiopaque line resembling cystic lesions (6,7). 

As mentioned in previous studies, in the cases of periradicular radiolucent lesions, sufficient biomechanical cleaning of the root canal system is the most critical factor for healing. It has been demonstrated that in these cases, non-surgical root canal therapy should be the first line of treatment (2) and approximately 74% of 42 endodontically treated teeth in one study showed bony healing within their large periradicular lesions (5). While some studies have shown no difference between large and small lesions’ healing ability (8), according to Calişkan the prognosis for large periradicular lesions is lower (5).

Permanent restoration within two weeks of RCT also contributed to periradicular healing, as several studies have shown that an adequate coronal restoration-placed as soon as possible after RCT-plays an important role in the outcome of endodontic therapy (9-11).

This patient was a young healthy subject and these factors will contribute to successful radiographical and clinical healing; previous studies have showed that the patient’s general health may have an influence on the healing process in periradicular lesions (2). Although the number of appointments (one-visit versus two-visit) for root canal therapy was one of the most controversial issues in endodontics for years, a Cochrane systematic review in 2008 revealed that there is no significant difference between single and multiple visits in the radiologic success of RCT (12). This case was treated in one visit, confirming that one-visit RCT can have successful results (13).

Radiographic changes such as the increase in density of the lesion and trabecular regeneration, confirmed healing in addition to the absence of signs and symptoms. However it is difficult to be sure of complete healing with conventional radiographic techniques.

## CONCLUSION

In the present case, single visit root canal therapy without any intracanal medicament, proved successful in promoting healing of a large cyst-like periradicular lesion. The result confirms previous reports demonstrating that even large periradicular lesions can respond successfully to non-surgical single-visit endodontic treatment.
